# Atypical Kawasaki Disease in an Adolescent with Multivisceral Involvement

**DOI:** 10.1155/2021/8941847

**Published:** 2021-07-27

**Authors:** Zohair El Haddar, Aziza El Ouali, Ayad Ghanam, Maria Rkain, Noufissa Benajiba, Abdeladim Babakhouya

**Affiliations:** ^1^Department of Pediatrics, Mohammed VI University Hospital, Oujda, Morocco; ^2^Faculty of Medicine and Pharmacy, Mohammed Ist University, Oujda, Morocco

## Abstract

Kawasaki disease (KD) is a vasculitis mostly seen in children aged less than 5 years. It can involve different organs and tissues. Its diagnosis is based on the clinical criteria of the American Heart Association (AHA). We report a case of a Moroccan adolescent with an atypical presentation of KD initially treated as typhoid fever. Gastrointestinal, renal, and pulmonary signs were the main clinical findings that made the diagnosis of KD challenging and delayed. The consequence was a severe cardiac damage with myocarditis and coronary artery dilation. KD is uncommon in adolescents, and it is important to recognize the atypical forms and the different presentations of KD in order to prevent the delay of diagnosis and treatment, and hence the cardiac complications.

## 1. Introduction

Kawasaki disease (KD) is a vasculitis of an unknown etiology first described in 1967 [1]. It usually affects children younger than 5 years. Small and medium arteries are the most concerned by KD which makes it the first cause of pediatric acquired heart disease [2]. Its diagnosis is based on the clinical findings of the American Heart Association (AHA) [2]. The KD can affect also adolescents and even adults; the clinical profile is different with a higher frequency of atypical presentations [3]. We report a case of a Moroccan adolescent presenting an atypical form of KD with gastrointestinal, renal, and pulmonary involvement.

## 2. Case Summary

**Figure 1:**
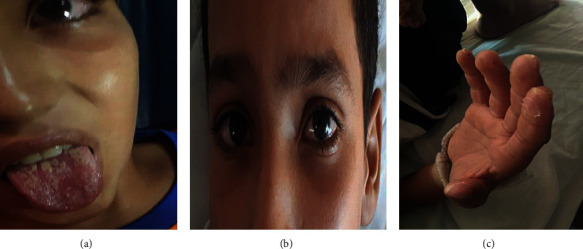
Clinical symptoms in our patient. (a) Coated tongue. (b) Bilateral ocular redness (uveitis). (c) Periungual desquamation.

**Figure 2:**
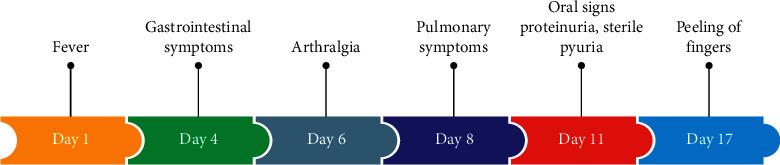
The timeline of the clinical case.

An 11-year-old boy was referred in January 2019 to our Department of Pediatrics because of a prolonged fever lasting 10 days associated with gastrointestinal signs resistant to antibiotics. There were no known allergies and personal or family history of prolonged fever or any systemic disease. The patient was febrile with deterioration in his general condition. Notably, acute abdominal pain associated with vomiting and diarrhea was reported one week before his admission. Therefore, the diagnosis of typhoid fever was considered, and antibiotic therapy including intravenous ceftriaxone 100 mg/kg/day was started without any favorable improvement. In addition, arthralgia, hives, cough, and chest pain were also described. On physical examination, the child was asthenic with tachypnea and tachycardia. Abdominal palpation revealed a periumbilical tenderness. Furthermore, a skin rash on the chest area which had disappeared 2 days after appearing was noted. Bilateral ocular redness without any suppuration was also noticed. Moreover, cracked lips, a coated tongue, and pharyngitis (Figure 1), associated to nonpitting edema of the right foot and ankles were identified. Remarkably, periungual desquamation appeared few days after his admission (see timeline in Figure 2).

**Figure 3:**
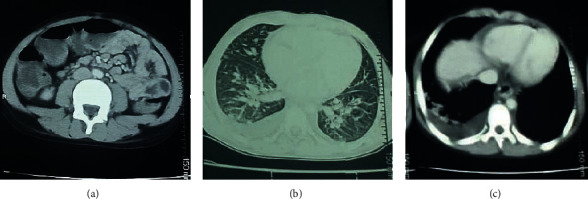
CT scan images showing (a) inflammation of the small bowel, air-fluid levels, reactional mesenteric lymphadenopathy, and (b), (c) right-sided pleurisy with bilateral pulmonary consolidation.

Biologically, a hypochromic microcytic anemia with hemoglobin at 9 g/dl, lymphopenia at 810/mm^3^, a high level of C-reactive protein (CRP) at 200 mg/L, a high erythrocyte sedimentation rate (ESR) at 64 mm/h, a normal platelet count at 245 g/l, and hypoalbuminemia at 26 g/l were found. The blood culture and procalcitonin level were normal; additionally, the serologies for hepatitis A, B, C, cytomegalovirus (CMV), Epstein–Barr Virus (EBV), human immunodeficiency virus (HIV), typhoid fever, *Mycoplasma pneumoniae*, leptospirosis, brucellosis, rickettsia, tuberculosis, and the hemophagocytic syndrome tests were without abnormalities. Moderate proteinuria at 412 mg/d (10.3 mg/kg/day) with sterile pyuria was the main renal finding. AST and ALT were <30UI/L, and complement 3 (C3), complement 4 (C4), and antinuclear antibodies were also within the normal range. However, the immunoglobulin E (IgE) level was abnormally so high at 1186 UI/ml. The radiological investigations were performed with a cerebral, thoracic, and abdominopelvic computed tomography (CTAP CT) scan; the results showed inflammation of the small bowel, air-fluid levels, reactional mesenteric lymphadenopathy, and a right-sided pleurisy with bilateral pulmonary consolidation (Figure 3).

**Figure 4:**
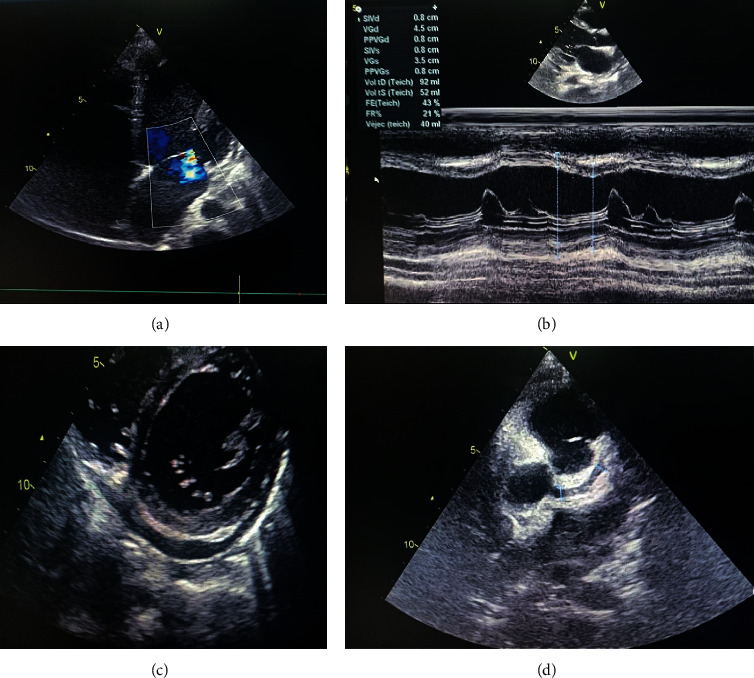
Echocardiographic images showing (a) mitral regurgitation, (b) low left ventricular ejection fraction at 43%, (c) pericardial effusion, and (d) 4 mm coronary dilatation (*Z* score +4).

His cardiac evaluation using transthoracic echocardiogram revealed a myocarditis, pericardial effusion, mitral regurgitation, 4 mm coronary dilatation (*Z* score +4), and a low left ventricular ejection fraction at 43% (Figure 4).

Sinus tachycardia and left ventricular hypertrophy at the electrocardiography were seen. Besides, cardiac troponin was high at 397.5 ng/ml with an increased brain natriuretic peptide (BNP) at 633 pg/ml. A therapeutic protocol based on intravenous immunoglobulin (IVIg) at a dose of 2 g/kg associated with acetylsalicylic acid 100 mg/kg/day during the acute phase was started 17 days after the onset of the symptoms, then after 5 days acetylsalicylic acid 5 mg/kg/day (antiaggregating dose) per os during 3 months.

During his follow-up, the proteinuria disappeared after few days, and there were no pulmonary lesions on the CT scan control. The coronary dilation has changed from 4 mm to 2.5 mm. The CT scan and the echocardiography were both performed 3 months after onset of treatment.

## 3. Discussion

Children under the age of 5 years are the most commonly affected age group by KD [2]. It was reported that adolescents represent only 1–7% of the patients [3–6]. Importantly and in a recent study, only 5.3% of children diagnosed with KD were older than 10 years [3]. In our case, the patient was 11 years old. The diagnosis of KD in its typical form is based on the clinical features of the American Heart Association (AHA) which include prolonged fever and four of the following criteria: polymorphous exanthem, extremity changes, mucosal changes involving the lips and oral cavity, bilateral bulbar conjunctival injection, and unilateral cervical lymphadenopathy [2]. Otherwise, the diagnosis of the atypical forms remains challenging for clinicians and paediatricians [3]. Up to date and to the best of our knowledge, this is the first case with concomitant pulmonary, gastrointestinal, and renal involvement to be described.

According to AHA recommendations, atypical KD is considered in children with prolonged fever and at least 2 compatible clinical criteria associated with echocardiographic findings and biological signs including late thrombocytosis, anemia, hypoalbuminemia <30 g/L, elevated alanine aminotransferase, WBC ≥15,000/mm^3^, and pyuria [2].

The echocardiographic findings supporting the diagnosis of the atypical KD are as follows.

- *Z* score of right coronary artery or left anterior descending coronary artery ≥2.5
- Coronary artery aneurysm
- Three or more other suggestive features including *Z* scores in the left anterior descending coronary artery or right coronary artery: 2–2.5, pericardial effusion, decreased left ventricular function, and mitral regurgitation [2]

Different gastrointestinal manifestations in KD have been reported in the literature; they vary from vomiting, abdominal pain, diarrhea, abdominal distension, paralytic ileus, hepatomegaly, jaundice, and hydrops of gallbladder to less frequent findings: pancreatitis, gastrointestinal obstruction, pseudoobstruction, sigmoid colitis, and sclerosing cholangitis [7]. The prevalence of gastrointestinal clinical symptoms in KD is unknown [7, 8]. Miyake et al. and Fabi et al. reported, respectively, a prevalence of 23% and 35% in their studies [8, 9], and a high percentage was found in radiological examinations, especially of the gallbladder [10].

The gastrointestinal involvement in KD can lead to a delay of diagnosis and treatment, unnecessary surgical intervention, more cardiac complications, and more immunoglobulins resistance [8, 9]. Sterile pyuria and proteinuria are the renal features in our observation and the two most common reported renal symptoms in KD [11]. The kidney inflammation may be a consequence of renal vasculitis or immune-related mechanisms [11]. Different renal disorders have been described in the literature. Chuang et al. reported an incidence of 28% of acute kidney injuries [12]. Other less common features can be found in KD: hemolytic-uremic syndrome, nephrotic syndrome, acute renal failure, renal scars, renal tubular abnormalities, immune complex-mediated nephropathy, and acute nephritic syndrome [11–16].

Pulmonary presentation of KD is exceptional. Singh et al. showed that only 1.83% of patients had a pulmonary involvement [17], while this percentage can reach 8.7% in adolescents [3]. The main symptoms are pleural effusion, pneumonia, pulmonary nodules, bronchopneumonia, and hydropneumothorax [18]. Recently, Arslanoglu Aydin et al. identified in their review of literature on pulmonary involvement in 20 KD patients with pleural effusions [18]. Patients and especially adolescents with pulmonary involvement due to KD may be more likely to have atypical KD and coronary artery abnormalities (CAA) due to diagnosis and therapeutic delay [3, 18]. The median interval between the onset of fever and the diagnosis of KD in adolescents is higher than younger children [3, 4, 19] which was in line with our case (17 days). Compared to younger children, the adolescents had a higher incidence of atypical KD and also CAA [3–5, 19].

## 4. Conclusion

KD is a multisystemic vasculitis that can involve multiple organs and tissues. The diagnosis in adolescents may be delayed especially if the atypical signs are the first to appear. It must be kept in mind in its atypical form to prevent cardiac complications and especially coronary artery abnormalities.

## Data Availability

The patient's data used to support the findings of this study can be retrieved from the archives of the Department of Pediatrics at the Mohammed VI University Hospital of Oujda, Morocco.
